# Enhanced Responsivity of Photodetectors Realized via Impact Ionization

**DOI:** 10.3390/s120201280

**Published:** 2012-01-31

**Authors:** Ji Yu, Chong-Xin Shan, Qian Qiao, Xiu-Hua Xie, Shuang-Peng Wang, Zhen-Zhong Zhang, De-Zhen Shen

**Affiliations:** 1 State Key Laboratory of Luminescence and Applications, Changchun Institute of Optics, Fine Mechanics and Physics, Chinese Academy of Sciences, Changchun 130033, China; E-Mails: yuji4268@163.com (J.Y.); qiaoqian1985@yahoo.com.cn (Q.Q.); xhxie001@126.com (X.-H.X.); wsp0510@163.com (S.P.W.); exciton@163.com (Z.Z.Z.); shendz@ciomp.ac.cn (D.Z.S.); 2 Graduate University of the Chinese Academy of Sciences, Beijing 100049, China

**Keywords:** photodetector, responsivity, impact ionization

## Abstract

To increase the responsivity is one of the vital issues for a photodetector. By employing ZnO as a representative material of ultraviolet photodetectors and Si as a representative material of visible photodetectors, an impact ionization process, in which additional carriers can be generated in an insulating layer at a relatively large electric field, has been employed to increase the responsivity of a semiconductor photodetector. It is found that the responsivity of the photodetectors can be enhanced by tens of times via this impact ionization process. The results reported in this paper provide a general route to enhance the responsivity of a photodetector, thus may represent a step towards high-performance photodetectors.

## 1. Introduction

Photodetectors are sensors of light or other electromagnetic energy, and they have a variety of applications including image sensing, communications, environmental monitoring, chemical/biological sensing, remote control, *etc.* [1–9]. To realize these applications, photodetectors with high performance are always a necessity. Responsivity is one of the most important parameters that determine the performance of a photodetector, which is defined as the photocurrent generated in a photodetector under the illumination of a light signal divided by the incident optical power [10]. If a general route can be found to enhance the responsivity of a photodetector, the significance of this route will be self-evident.

It is accepted that under relatively high electric field, the electrons and holes in a condensed matter will be accelerated and gain much kinetic energy. The accelerated carriers may impact with the atoms in the lattice of the host materials, and lose their energy by exciting electrons in the valence band of the host materials to the conduction band, thus leaving free holes. The excited electrons and holes can also be accelerated and impact with the host lattice to excite additional carriers [8,11,12]. The above process is called impact ionization. By using the generated electrons and holes, lasing and light-emitting devices have been realized in zinc oxide (ZnO) based materials [11–15]. One can speculate that if the electrons and holes generated through the impact ionization process can be used in photodetectors, the responsivity of the photodetector could be enhanced greatly. Nevertheless, although there have been some reports on photodetectors employing impact ionization [16–18], a systematic study of the responsivity enhancement of a photodetector via impact ionization process is still absent.

In this paper, a general route to enhance the responsivity of a photodetector employing the impact ionization process has been proposed and demonstrated by employing ZnO as a representative material of ultraviolet (UV) photodetectors and silicon (Si) as a representative material of visible photodetectors. It is found that because of the additional carriers generated in the impact ionization process, the responsivity of photodetectors can be improved significantly.

## 2. Experimental Process

The ZnO film employed as the active layer of UV photodetector was grown on *c*-plane sapphire by a sol-gel method. Details of the synthesis and deposition process can be found elsewhere [19]. The Si employed as the active layer of visible photodetector was commercial-available silicon wafer. A MgO layer was deposited onto the ZnO film and Si wafer in a magnetron sputtering equipment acting as the insulation layer for the impact ionization process. The growth conditions of the MgO layer were as follows: the background vacuum in the growth chamber was 2.0 × 10^−3^ Pa. The temperature was fixed at 300 °C and the pressure set at 1.0 Pa during the sputtering process. Then a thin Au layer was evaporated onto the MgO films using a vacuum evaporation method, and during the evaporation, the pressure in the chamber was fixed at 2.0 × 10^−3^ Pa. Interdigital electrodes were configured via a photolithography and wet etching process. The devices are referred to as metal-insulator-semiconductor-insulator-metal (MISIM) structured photodetector in the following text. For comparison, interdigital Au electrodes were also patterned directly onto the ZnO and Si without the MgO insulation layer in the same procedure, and the devices are referred to as metal-semiconductor-metal (MSM) structured photodetectors in the following text. The absorption spectrum of the ZnO film was studied in a Shimadzu UV-3101 PC scanning spectrophotometer. Photoluminescence (PL) spectrum of the ZnO film was recorded in a JY-630 micro-Raman spectrometer using the 325 nm line of a He-Cd laser as the excitation source. The electrical characteristics of the photodetectors were measured using a Lakeshore 7707 Hall measurement system. The photoresponse of the photodetectors was measured in a SPEX scanning monochromator employing a 150 W Xe lamp as the illumination source.

## 3. Experimental Results and Discussion

**Figure 1.**
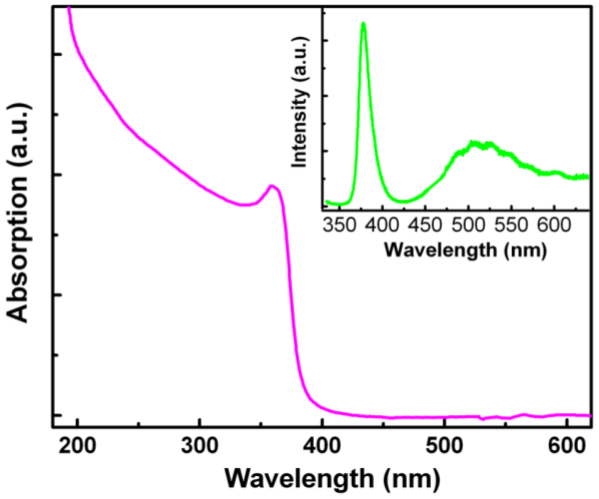
Room temperature absorption spectrum of the ZnO film, and the inset shows PL spectrum of the ZnO film.

Figure 1 shows the absorption spectrum of the ZnO film. It can be seen that the absorption edge locates at around 380 nm, which corresponds to the excitonic absorption of ZnO. The absorption in the UV region is significantly stronger than that in the visible spectrum region, which is essential for its application in high-performance UV photodetectors. The inset of Figure 1 shows room temperature PL spectrum of the ZnO film. A strong emission at around 379 nm and a broad emission at about 516 nm can be observed from the figure, both of which are typical emissions of ZnO, the former is the near-band-edge emission, while the latter is the deep-level emission.

**Figure 2.**
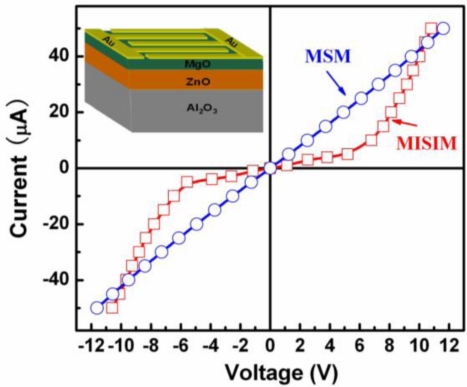
Current-voltage characteristics of the ZnO based MISIM and MSM photodetectors under dark condition. The inset shows a schematic diagram of the ZnO MISIM-structured photodetector.

The inset of Figure 2 shows the schematic illustration of the ZnO MISIM structured photodetector. The thickness of the ZnO film is about 180 nm, and that of the MgO layer is about 60 nm. The Au fingers of the interdigital electrodes are 500 μm in length and 5 μm in width, and the inter-electrode spacing is 10 μm. The current-voltage (*I–V*) characteristics of the ZnO based MISIM and MSM photodetectors under dark conditions are illustrated in Figure 2. Compared with the *I–V* curve of the MSM photodetector, an obvious rectifying behavior can be observed from that of the MISIM one, which indicates that Schottky contact has been obtained by inserting the MgO insulator layer. Note that the dark current of the MISIM photodetector is smaller than that of the MSM photodetector when the applied voltage is below 10 V, while the situation is reversed when the applied voltage is above 10 V. The above phenomenon may result from the carrier multiplication due to the impact ionization process in the MgO layer under a high electric field, which will be detailed in the following text.

**Figure 3.**
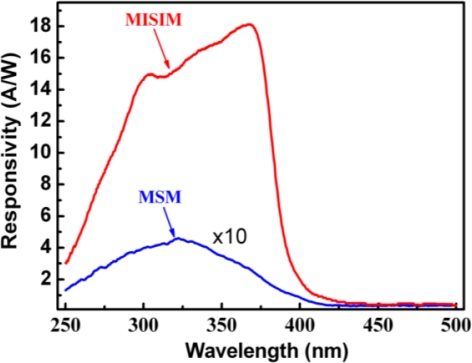
Photoresponse of the MISIM and MSM structured ZnO photodetectors at 24 V bias. Note that the responsivity of the MSM photodetector has been magnified by 10 times.

**Figure 4.**
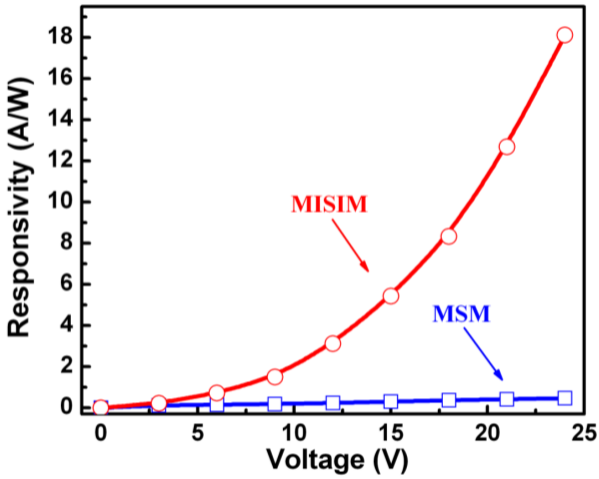
Responsivity of the ZnO MISIM and MSM photodetectors as a function of the applied bias.

The response spectra of the ZnO based MISIM and MSM structured photodetectors under 24 V bias are shown in Figure 3. The maximum responsivity of the MISIM photodetector is about 18.1 AW^−1^, which is almost 38 times larger than that of the MSM one (0.46 AW^−1^). The responsivity of the ZnO based MISIM and MSM photodetectors as a function of the applied bias is illustrated in Figure 4. We note that the responsivity of the MSM photodetector shows a linear dependence on the applied bias, while that of the MISIM photodetector increases exponentially with increasing applied bias. The external quantum efficiency (EQE) *η* of a photodetector can be expressed by the following formula [8]:

(1) $$\eta =\frac{I_{p}/e}{P_{\text{opt}}/h\nu }$$

where *I*_p_ is the photogenerated current under the illumination of the incident light, *e* is the elementary charge of electrons, *P*_opt_ is the incident optical power, *hν* is the photon energy. The maximum EQE of the ZnO MSM structured photodetector at 24 V bias determined from Equation (1) is 177%, while that of the MISIM photodetector is as high as 6,150%, which indicates that the MISIM structured photodetector has a great optical gain.

**Figure 5.**
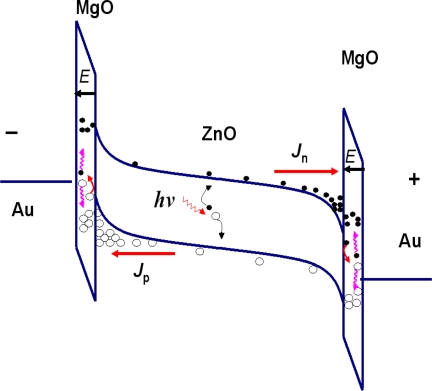
The schematic illustration of the band alignment of the MISIM structured ZnO photodetector showing the occurrence of carrier multiplication via the impact ionization process.

The origin of the optical gain for the ZnO MISIM photodetector can be ascribed to the impact ionization process in the MgO layer. To understand the mechanism better, the bandgap alignment of the MISIM structure is shown in Figure 5. Under dark conditions, the residual carriers in the ZnO film will drift towards the interdigital electrodes under the drive of bias. Note that they have to pass through the MgO insulation layer to reach the electrodes. Due to its dielectric nature, most of the voltage will be applied onto the MgO insulation layer. Then the electric field in the MgO layer will be as high as 0.8 × 10^6^ V/cm at 10 V bias considering the thickness of MgO is only about 60 nm. Under such a high electric field, the residual carriers in the ZnO layer that can enter into the MgO layer will gain much kinetic energy, and they will impact with the lattice of the MgO layer, thus the electrons in the valence band of MgO will be excited, and free holes are left. In this way, additional electrons and holes are generated [20]. The above process is known as impact ionization process. These additional carriers will also be swept to the corresponding electrodes by the bias. As a result, the number of carriers that can be collected by the electrodes is increased greatly. This is the reason why the dark current of the MISIM structure is larger than that of the MSM structure when the bias is larger than 10 V. While at small bias (smaller than 10 V in our case), the electric field applied onto the MgO layer is not large enough to reach the impact ionization threshold of MgO (about 10^6^ V/cm [18]), then impact ionization process mentioned above will not occur, thus no additional carriers will be generated. Under such circumstance, the MgO layer behaves like an insulator that hinders the transportation of electrons and holes to their corresponding electrodes, thus the dark current of the MISIM structure is smaller than that of the MSM structure, as shown in Figure 2. The above effect of an insulation layer on the reduction of dark current has been well studied in semiconductor photodetectors [21,22].

Here we show that by virtue of the impact ionization process, the responsivity of a photodetector can be enhanced greatly. The situation is similar to that mentioned above. Under the illumination of the UV light whose energy is larger than the bandgap of ZnO, free electrons and holes will be generated in the ZnO layer. Under bias voltage, the photogenerated electrons and holes will be drifted towards their corresponding electrodes. When passing through the MgO insulation layer, the carriers will gain much kinetic energy, and will impact with the lattice of the MgO layer, thus exciting additional electrons and holes. As a result, the number of carriers that can be collected by the electrodes increases greatly, then the responsivity of the MISIM structured photodetector is enhanced. At larger bias, more carriers will be generated through this impact ionization process, then the responsivity of the MISIM structured photodetector increases exponentially with the applied bias, as shown in Figure 4. While for the MSM structured photodetector, such a carrier multiplication process is absent, thus its responsivity is much smaller than that of the MISIM one, and its responsivity increases linearly with the applied voltage.

## 4. Conclusions

In summary, by employing ZnO as a representative material of ultraviolet photodetectors and Si as a representative material of visible photodetectors, metal-insulator-semiconductor-insulator-metal structured photodetectors have been fabricated. It is found that the responsivity of the ZnO based ultraviolet photodetector has been increased by 38 times, and that of the Si based visible photodetector increased by 23 times compared with their metal-semiconductor-metal structured counterparts. The mechanism for the enhancement is caused by the carrier-multiplication in the insulation layer due to the impact ionization process. The results reported in this paper provide a general route to enhance the responsivity of a photodetector, thus may represent a step towards high performance photodetectors.

## Figures and Tables

**Figure 6.**
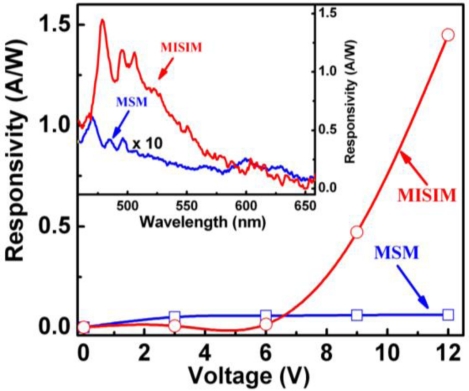
Responsivity of the Si based MISIM and MSM photodetectors as a function of the applied bias. The inset shows the photoresponse of the MISIM and MSM structured Si photodetectors measured at 12 V bias (note that the responsivity of the MSM photodetector has been magnified by 10 times).
